# Is Psychoanalysis a Folk Psychology?

**DOI:** 10.3389/fpsyg.2013.00135

**Published:** 2013-03-22

**Authors:** Mathieu Arminjon

**Affiliations:** ^1^Département Universitaire de Psychiatrie, Faculté de Médecine, Université de GenèveGenève, Switzerland; ^2^Agalma FoundationGenève, Switzerland

**Keywords:** neuropsychoanalysis, hermeneutics, reasons, causes, folk psychology, interpretation

## Abstract

Even as the neuro-psychoanalytic field has matured, from a naturalist point of view, the epistemological status of Freudian interpretations still remains problematic at a naturalist point of view. As a result of the resurgence of hermeneutics, the claim has been made that psychoanalysis is an extension of folk psychology. For these “extensionists,” asking psychoanalysis to prove its interpretations would be as absurd as demanding the proofs of the scientific accuracy of folk psychology. I propose to show how Dennett’s theory of the intentional stance allows us to defend an extensionist position while sparing us certain hermeneutic difficulties. In conclusion, I will consider how Shevrin et al. (1996) experiments could turn extensionist conceptual considerations into experimentally testable issues.

## Introduction

In spite of the recent data that neuropsychoanalysis has brought forward in favor of some aspects of the Freudian theory (Arminjon, 2011), the status of interpretations in psychoanalysis, their accuracy and scientific vindication, remains problematic. Grünbaum’s (1984, 1986) critique of Freud’s tally argument, calling for extra-clinical proofs, still governs the shape of the debate. Nonetheless, a few decades ago we began to witness a renewed interest in commonsense or folk psychology. Several theorists in the field saw it as a means to pursue the hermeneutics defense of the autonomous scientific status of psychoanalysis. In a broad outline, the rationale consists of claiming that no one would deny the accuracy of our daily folk psychological explanations of our own and others’ mental states. We spontaneously attribute reasons to them to causally explain their behaviors and mental states. In the same vein, psychoanalysis could be nothing more than an *extension of folk psychology*. In which case, asking to prove how accurate psychoanalytic interpretation would be as absurd as asking people to give proofs of the scientific reliability of folk psychology.

My aim here consists in specifying to what extent we can conceptually say that psychoanalysis is an extension of folk psychology. To reach that goal, I will try to uncover the way in which the hermeneutic reading of psychoanalysis put the debate in terms of a defense of the distinction between causes and reasons in an effort to free psychoanalysis from the burden of having to prove itself. After having considered Grünbaum’s main arguments against this position, and especially against the hermeneutic reading that would be acausal, I will show how extensionism referred to folk psychology in order to return Grünbaum’s rationale against his own arguments. I will defend the claim that both Grünbaum’s and the extensionists’ positions are misleading. I will do so by referring to Dennett’s intentional stance, since it provides a theory of interpretation that allows determining in what extent folk psychology can be scientifically used. From a naturalist^1^point of view, I will generally defend the idea that the intentional stance thus can be said to be a *causal-hermeneutics*. On this basis, I will lastly expose how the seminal experiments performed by Shevrin et al. (1996) may allow turning these conceptual considerations into a testable issue.

## The Hermeneuticized Freud

In the twentieth century, hermeneutics^2^ emancipated itself from its exclusive location in the field of biblical exegesis and became the designation for the general study of the theory and practice of interpretation. Dilthey posited hermeneutics as the salient epistemic boundary that divided the method of the natural science *explaining* phenomena in terms of blind natural causes and the Humanities, which give us an understanding of human actions by reconstituting their inner reasons throughout an empathic process. The latter would determine the scientifically acceptable way it would be possible to experiment in the fields of history, sociology, or psychology.

In line with the hermeneutics tradition, modern hermeneuts (Jasper, Ricoeur, Habermas…) considered psychoanalysis, contra experimental psychology, as the interpretative science par excellence. To Ricoeur (1970), for instance, There is no observational fact in psychoanalysis, only interpretations! If experimental psychology gives the causes of actions, thus misses their intentional or motivational dimension, psychoanalysis gives a “deep” understanding of their reasons. The causes and reasons distinction would firstly vindicate psychoanalysis as an autonomous science. Secondly, it would justify Ricoeur’s claim that attempting to ground psychoanalysis on a natural science basis is *a priori* useless, not to say an epistemological nonsense! As a matter of fact, the hermeneutical tradition is tied with the idea that the interpretation of a text, an action, a thought, etc., depends on the encounter between an interpreter and a text, a historical event or an action. Interpretation is not the literal reconstruction of a genuine intention, but the attempt to make an intention comprehensible at the light of the interpreter’s historical and idiosyncratic expectancies. In other words, an interpretation is a never-ending negotiation process intervening between the interpreter and the interpretans’ situated perspectives, as a “fusion of their horizons,” as Gadamer (1997) would put it. In a sense, hermeneutics is in line with the Quine (1960) inscrutability of the interpretation’s referent. No deep or hidden meaning would constitute an objective fact that would foreclose on any further conflict of interpretation. Thus several interpretations, even contradictory ones, might relevantly and practically account for the same texts, actions, etc.

The French psychoanalyst Viderman (1970) applied the hermeneutical program and the indeterminacy of the interpretation at its heart to the difficulties inherent to Freudian interpretations. Indeed, most of the Freudian interpretations are not only determined by the “psychic material” provided by patients, but also by Freud’s modification of the patients’ narrative, by which Viderman meant the inversions or inventions of meanings that Freud introduced. If this is so, why then would Freud claim that he was only reconstructing objective causes of symptoms? To Viderman, Freud’s position would have been more warranted if he had claimed that psychoanalysis was simply about constructing healing fictions or narratives. If nothing really counts as an objective fact of interpretation, then the final analyst’s interpretation “does not have reconstructed a historical scene, but builds a hypothetical scene, perfectly consistent, where historical elements constitute magnetizing points that yield cohesion to the posterior fantasy” (Viderman, 1970, my translation). In other words, interpretations rightness would not be indexed on its accuracy, i.e., its capacity to enlighten mental objective referents, yet on its curative efficacy.

## Grünbaum on Causes, Reasons, and Interpretation

The difficulties tied with the epistemological status of interpretation in psychoanalysis are at the heart of Grünbaum’s essential critique of psychoanalysis. I propose to consider two of his arguments. The first is an attack on what is, at least for Grünbaum (1984, 1986), the sole argument Freud proposed to vindicate psychoanalytic interpretations. The second (Grünbaum, 1984, 1986, 2004) is a rejection of the hermeneutical distinction between causes and reasons and its epistemological implications.

To Grünbaum Freud only provided one argument for validating interpretations. The “tally argument,” as Grünbaum names it, consists in claiming that «[Patient’s] Conflicts will only be successfully solved and his resistances overcome if the anticipatory ideas he is given [the psychoanalytic interpretation] tally with what is real in him» (Freud, 1963).

Grünbaum (1984, 1986) convincingly shows that a curative effect can only be validated in comparison to group controls. Thus, in the absence of such extra-clinical data, to impute the therapeutic effects to the psychoanalytic interpretation is no more accurate than to impute it to suggestion, spontaneous remission, or placebo. To prove the validity of the psychoanalytic cure, Freud would have to have submitted it to the kind of comparisons between group controls, as it is usually required in medicine.

As we can see, the hermeneutic reading of psychoanalysis attempts to immunize psychoanalysis against this kind of attack. In Grünbaum’s terms, in defending the causes-reasons distinctions, hermeneutics has tried “(a) to free the study of human ideation from the evidential burdens of the standards empirical sciences and (b) to draw an ontological boundary line between mental and other natural processes so as to strengthen the case for (a)” (Grünbaum, 2004). Thus, the causes-reasons distinction is revealed as nothing more than a strategy to make the extra-clinical argument, as a necessary condition for validating an interpretation, to be *a priori* absurd.

Thus, the second of Grünbaum’s (1984, 1986) attacks to which I here refer to consists in pointing out acausality as an invalidating limit to the hermeneutic reading of Freud’s theory. Grünbaum’s reasoning implies three arguments: (1) the principles of hermeneutic interpretations make a non-technical use of the concept of cause and reason, especially in reducing causality to physics events. (2) Adopting the physicalist myth might lead us to consider reasons as distinct from causes whereas the practical syllogism and the liberal definition of causality, yield the criteria of the causal properties of reasons. (3) On this basis, the hermeneutic advocates wrongly claim that Freud’s explanations determine not physical causes but mental reasons, whereas in reality Freud’s explanations simply do not meet the elementary criteria of the practical syllogism. Grünbaum’s argument for the causal relevancy of the practical syllogism to the question of causality goes like this:

The relation of causal relevance between an antecedent X and an outcome Y:
X may be a physical, mental, or psychophysical cause if it makes a difference to the occurrence of Y and affect the incidence of Y.
Explications in terms of reasons are normally in conformity with the practical syllogism:
X carries out an action Z because he *desires* to achieve a goal Y, and also *believes* that doing Z will achieve Y. Thus the desire-cum-belief set supplies “the reason” for doing Z. But psychoanalytic explanations do not conform to this model; they fail, in other words, to justify themselves with reference to the practical syllogism.

“Unconscious motives do not constitute such “reasons,” because classical psychoanalytic explanations typically do not event conform to the practical syllogism. It turns out that the explanatory motives do not include an unconscious belief that the explained behavior is a means of realizing the repressed aim. Therefore, such behavior fails to be a species of intend action, although the impulse that instigates it can be said to be a repressed aim or “intention” (Grünbaum, 1986).

To sum up, then, the supporters of hermeneutics, by refusing, *a priori*, to reduce interpretation to explanation within the naturalistic framework, do offer the advantage of highlighting the heuristic nature of the psychoanalytic interpretative framework. As such, they are constructing the premise of the theory that would identify psychoanalysis with an extension of folk psychology. But as far as this epistemological distinction is fruitful in leading to clarifying the composite nature of psychoanalysis’s causally irreducible discourses, identifying it with a Dilthey-like set of understandings, rather than physical causes, it also, necessarily, leads to Grünbaum’s accusation of acausality (acausal-hermeneutics). If interpretations are not causally linked, by one means or another, to a causal process, how could they elicit any positive effects on the ego, not to say on the brain? And if no fact of any kind yields any constrains or validation of the interpretative course, a reason justified inquiry is bound to fall to relativism, the impossibility to weigh reasons one against the other or, in other words, relativism.

Before analyzing the relevance of Grünbaum’s arguments against the cause-reasons distinction, I propose to expose how extensionists paradoxically adopted the latter, in order to defend the autonomy of psychoanalysis. In other words, I want to show how they cleverly accept Grünbaum’s statement on the causal status of reasons and turn it against him in order to rehabilitate the hermeneutics’ defense of the epistemological autonomy of psychoanalysis.

## The Extensionist Response

What I am calling extensionism is defined as the theory according to which psychoanalysis would be an extension of folk psychology. To understand it, we can refer to Thomas Nagel’s (1994) classic justification of it. Nagel interprets Freud’s philosophy of mind to consist in a threefold claim: (1) that conscious mental states are physical states of the brain: (2) nevertheless, that some other states are analogous to conscious ones, i.e., they induce the same psychological effects, but are unconscious, and thus we can apply to them the psychological terms or intentional idiom we use for conscious states: and (3) consequently, the problem of psychoanalysis does not concern the ontological nature of “unconscious mental state^3^,” but turns on an epistemic question: what is the utility of such an extension of folk psychology? And, on top of it, what evidence supports it?

Given that we define folk psychology by reference to the way people spontaneously understand, explain, and predict other’s behaviors and mental states on the basis of beliefs and desires attributions, then it is evident from psychoanalytic terminology that we are still engaged, here, with an extended folk psychology. What does the term “extension” means here? To Edelson (1988), for instance, psychoanalysis does not extend folk psychology conceptually, e.g., bringing out new propositional attitudes for instance. The point is that most of the psychoanalytic concepts such as unconscious, primary processes, repression, ambivalence, etc., are commonly taken to refer to psychological mechanisms, while at a clinical level, they represent the tools allowing to literally extending the range of processes that are explainable by the means of folk psychology.

Extensionism can be seen as a mid-position between the hermeneutics defense of psychoanalysis’ scientific autonomy and Grünbaum’s realist conception of the causal status of the practical syllogism. As a matter of fact, if ascribing reasons is a sort of assignment of causes, i.e., if they describe the mental states that really determine one’s behavior, then Hopkins (1988) remark has a lot of force: “… why controls should be required for psychoanalytic but not common sense judgments on the role of motives. If commonsense cogency as to causal relevance is in question, then surely, it seems, commonsense practices (or their extension) might suffice for it.”

Indeed, nobody would require scientific validation for the mental ascriptions of ordinary life. If we are really set on denying the causal relevancy of the reasons we ascribe, then not only Freudian interpretations, but also all our mind readings attempts become groundless. As a consequence, the justification of interpretations would not rest on empirical facts but would be a matter of “holistic coherence^4^.” Since “commonsense reasoning […] suits the psychological properties of persons, which are rarely uniformly repeated but always pervasively and non-coincidentally related in content” (Hopkins, 1988), an interpretation is confirmed or disconfirmed in function of its capacity to appear as coherent. If it enters into a cogent connection with the others wishes, beliefs, actions, etc., the analyst can ascribe to the patients, the interpretation the analyst presents is accurate. The same rationale would be applicable to the therapeutic success. If it is “part of commonsense psychology that motives and wishes can be modified by awareness and thought” (Hopkins, 1988), thus the therapeutic success would take part to the holistic validation procedure of interpretations.

## The Aliquis Forgetting

But what about Grünbaum’s argument according to which the classical psychoanalytic explanations would fail at conforming to the practical syllogism? Before showing in which extent extensionism fails at overcoming the main limits of the acausal-hermeneutics, I propose to attack Grünbaum here, since I claim that it is possible to make psychoanalytic explanations conform to the practical syllogism, without doing violence to the main claims of psychoanalysis.

Consider the famous Aliquis forgetting Grünbaum’s analysis is generally based on. In a classical case outlined in The Psychopathology of everyday Life (Freud, 1965), Freud tells of having met a young academician on a train who, in the course of the discussion, forgot one word, “aliquis,” when he tried quoting Virgil. Freud hypothesized that something, an unconscious determinant, had induced the forgetting. After having questioned the scholar, Freud interprets that the word “aliquis” is very close to the word “liquid” and then has been repressed since the scholar is afraid his mistress might be pregnant (aliquis, liquid, period)^5^. According to Grünbaum (1984), “This well-known case fails to conform to the practical syllogism. For there is not a shred of evidence that the male subject underwent his memory lapse in the unconscious belief – however foolish – of thereby realizing (fulfilling) his desire (hope) that his paramour is not pregnant.”

According to him, psychoanalysis cannot yield the reasons of symptoms taken that Freudian classic explanations do not conform to the practical syllogism (X desires to do Y, and believes that Z achieves Y, thus X does Z). On that model, the aliquis case should be described as follows: X desires his paramour not to be pregnant, he believes that if he forgets her putative pregnancy, she won’t be pregnant, so he forgets her pregnancy or any word associatively linked to pregnancy. To Grünbaum, such a syllogism is invalid. The first reason why would be that the belief is logically “foolish.” And he is right, what an irrational belief it would be to think that if you forget something it will make it disappear! Here are the paradoxes of self-deception: how could one think to forget something without thinking of it? But this is precisely the kind of egregious irrationality Freud wanted to explain. Secondly, the explanatory motives would not, according to Grünbaum, include an unconscious belief that the behavior to be explained is a means of realizing the repressed aim. Let’s apply it to the aliquis case.

The Freudian concept of “compromise formation” is precisely the kind of notion that allows extending mental ascriptions. It posits that a symptom can be said to be the by-product of an inner conflict that partly expresses the warring tendencies of that conflict. If so, the forgetting of the word aliquis can be divided into two independent desire-cum-belief expressions, which both conform to the practical syllogism. On the one hand, the scholar desires to be responsible and believes that taking his mistress’s putative pregnancy seriously will allow him to cope with it. So he does so. On the other side, he desires not to be effected by bad news, and believes that avoiding thinking that his mistress is pregnant will preserve him from bad feelings, so he avoids any hint that would lead him to think of his mistress’ putative pregnancy.

Consequently, the forgetting may have two partial reasons, one tending to repress unpleasant thoughts, the other one tending to occupy the scholar’s attention. In a sense, the forgetting is the means by which the repressed aim is realized, and the fact that the scholar is conscious of his incapacity to recover the quote he is supposed to know by heart, is the way the other intention partly succeeds too. The problem with Grünbaum’s analysis consists in making the forgetting the illogical consequence of the desire-cum-belief conjunction, instead of considering the conflict as inducing a disturbance of thoughts that are decoupled from the conflict. Whatever Grünbaum thinks, this is clearly the conclusion Freud (1965) is driven to when considering the aliquis forgetting as induced by “the disturbance of a thought by an internal contradiction which arises from the repressed.”

## The Weakness of Extensionism and Virtues of Hermeneutics

As we can see it, whatever “foolish,” there are always “special stories” allowing reconstructing the reasons of weird actions, lapses, symptoms, etc. But even if it is right, in adopting the realist conception of folk psychology, to restore psychoanalysis’ autonomy and its independence from any kind of extra-clinical data, extensionits end up falling in the hermeneutical trap; psychoanalysis is still acausal if it fails to give us the extra-clinical proof that would allow it to avoid plunging into relativism.

In postulating the explicative autonomy of folk psychology and then claiming psychoanalysis is an extension of it, extensionists expect that psychoanalysis is secured, as folk psychology is, from being subject to controlled testing. But nothing, except an unquestioned but problematic matching, explains how folk psychological ascriptions match the psychological properties of persons. This is precisely the kind of question that the advocates of hermeneutics put to realism in their defense of the cause-reason dichotomy. But it is also the kind of miraculous matching eliminativists – those who denied any accuracy to folk psychology – refute. Indeed, the realist conception of folk psychology endorsed by Grünbaum, and thus by the extensionists, is hardly sustainable either under a first or a third-person perspective. Let’s consider some problems.

Firstly, since Wittgenstein, it is common to say that reasons can only be alleged by the agent. Whatever true or false, only the agent is able to articulate its action according to his beliefs and desires, otherwise he could not recognize himself as the subject of its own action. The realist conception of reasons mostly rests on the assumption that subjects must enjoy here a privilege of infallibility and exclusiveness in accessing their reasons to act. The point is that such infallibility is performative, not epistemic. Several experimental studies on the sense of agency – the way we come to think we are agents who intentionally plan, realize, and control our actions – have shown that subjects, in specific circumstances, will misattribute to themselves action they did not intended (Wegner and Wheatley, 1999). If these experiments do not definitely exclude the people sometimes act for reasons, they nevertheless challenge the folk psychological judgment according to which our sense of agency must be immune to error. On this basis several scholars have claimed, against the kind of realistic position Grünbaum endorses, that reasons are not causal but *ex post* facto narrative rationalizations or confabulations regarding actions, better than the real mechanisms that cause actions (Gazzaniga, 1985).

Thus, even if we do admit that the description of actions that conform to the practical syllogism are driven by reasons, there is nothing, at least from a first-person perspective, that pleads in favor of its causal status. As we will see, even from a third-person perspective, many intentional like behaviors are retrospectively explainable in terms of reasons, even if we know perfectly well that there are no internal psychological mechanisms such as reason in them.

Secondly, when Grünbaum (1984) rejects what he calls the hermeneutist’s non-technical use of the concept of cause, he acts as if the status of mental causation was not a philosophical issue. Yet, taking for granted the causal status of reason leads to pretending that the mind-body problem or that psychophysics causation is non-problematic. Borrowing from Kim (1989), we may say that physics is closed and complete (closure principle), i.e., each event is caused by a precedent causal physical event and nothing immaterial can have any causal efficacy. Thus, if a mental state M is said to be causal, to a materialist, it must be realized by a physical base P. Now imagine that M causes a physical event P′, we must suppose that it has been caused by an antecedent physical event too, in this case P, that is the physical base of M. If we claim that P’ has two causes, it is said to be overdetermined. But according to the closure of physics, P is sufficient for P’ to occur. Thus, claiming that M is causal is superfluous or indicates the breakdown of Kim’s claim about physics, and the re-validation of dualism.

Consequently, Grünbaum (1984, 2004) might be right claiming that X is a cause if it makes a difference to the occurrence of Y. But does X, as a mental property, makes a difference in the occurrence of Y because of its physical basis or independently of it? To maintain his position, Grünbaum has to explain how a mental event can make a difference in the occurrence of Y without being instantiated on a physical basis and/or to what extent the mental “property” is not causally superfluous, i.e., what it metaphysically means that it can take part in the whole causal process determining a physical event. Of course Grünbaum’s assimilation of causes and reasons does present the advantage of being, as he puts it, applicable “alike in medicine, psychology, physics, sociology, and elsewhere” (Grünbaum, 2004). But where the mental properties are relevant, as in psychology, sociology and, sometime, in medicine, what proof is there that what is supposed to be mentally relevant here is not reducible to the underlying neurobiological, biological, and in fine, physical properties, that instantiate them? Instead of facing those difficulties, Grünbaum’s position consists in acting as if the causal status of mental processes were unproblematic and as if it would have received a well-accepted definitive solution. In other words, his liberal notion of the concepts of causes and reasons could be right, and it might turn out that physicalism is a fallacy, but without detailing what makes mental properties, and thus reasons, causally relevant, Grünbaum’s position appears as too indeterminate to seriously put physicalism in doubt.

Note that in claiming that Grünbaum and thus extentionists fail to motivate their causes-reasons identification, I do not dismiss the former’s general conclusion concerning hermeneutics’ incapacity to test its interpretations. Interpretations, to be scientifically acceptable, must be founded on facts, whatever they might be. Thus, what we have to deal with consists in addressing whether we can articulate the specificities of the hermeneutics theory of interpretation within the physicalist’s ontology. To do this, we have to define in what extent causal properties can determine facts of interpretation. That is precisely what the intentional stance theory (IS), by Dennett, will allow us to do.

## Is Psychology in the Eye of the Beholder?

We defined folk psychology as the way people spontaneously understand, explain, and predict other’s behaviors and mental states on the basis of beliefs and desires attributions. To realists, such as Grünbaum or the extensionists, these ascribed mental states refer to causal states in the persons. To eliminativists (for instance Churchland, 1981), folk psychological entities are non-existent. Nevertheless, some of the latter group, as Rorty (1988) for instance, acknowledge that folk psychology constitutes a conceptual schema, one among others, which only represents a normative framework collectively used to explain or evaluate others’ behaviors. Thus, folk psychology would not exist elsewhere than in the “eye of the beholders” and, consequently, would have no ontological nor explicative superiority to astrology, magical thinking, homeopathy, etc., except as being more widespread as an interpretative schema.

Mentioning Rorty’s position on folk psychology represents a good angle for introducing the IS that can be positioned at a midpoint between eliminativism and commonsense realism about mental states. If Rorty were right, there would be no way of distinguishing how folk psychology allows explaining others’ behaviors better than astrology. But we have means to show that the first works at predicting behaviors, while we have no such evidence in favor of the latter. Furthermore, the fact that intentional states might be observer-relative does not prevent folk psychology, at least not *a priori*, from referring to objective states, the nature of which we shall have to define. How can we show that it does refer to objective states that, in spite of their being in the eye of the beholder, make folk psychology testable, as compared to others interpretation schemas?

As Dennett (1981) proposes, imagine Martians landing on earth, who are able to predict humans’ future behaviors from the current physical states of the world and of their bodies (brains). Even if such a performance were epistemologically and technically possible, Martians, as Dennett puts it, would overlook the behavioral regularities humans successfully rely on to easily explain and predict others’ behaviors. Predictability is the core concept here. To emphasize its role, Dennett (1983) invites us to remember *A scandal in Bohemia*. In the Arthur Conan Doyle novel, Sherlock Holmes must find out a precious photography the well-identified blackmailer has hidden in a room. Holmes’ ploy simply rests on a conditional version of the practical syllogism that departs from the realist one:

– If the blackmailer desires to hide the compromising picture in a safe place.
– If he believes that, in the event of danger, removing the picture might increase the chances of preserving it.
– Then, in the event of danger, the blackmailer will remove it in a safer place.

The trap goes like this: Watson throws a smoke bomb in the house shouting fire. Holmes, hidden in the room, observes the blackmailer collecting the photograph and, consequently, revealing the hiding place.

According to Dennett, the intentional states’ referential status is a matter of fact. When Holmes predicts someone else’s behavior on the basis of attributions of beliefs and desires, his use of the folk psychology reveals the existence of objective behavioral regularities or patterns. The point is (1) that the objectivity of these patterns is retrospectively confirmed if, and only if, the prediction is successful; and (2) that the intentional states postulated are not causal or objective *per se*, but represent heuristics that make it possible to grasp these objective regularities. This second point is closely related to Rorty’s critics of the relativist nature of folk psychology, which otherwise appeared to be the dead end that doomed hermeneutics. Remember Lakoff and Johnson’s (1999) theory of color perception. It is false to say that colors exist neither as ontological properties in the world, nor in the mind as subjective states. Colors are “interactional properties,” testifying that the way human beholders, with their specific perceptive capacities, grasp objective physical regularities (wavelengths). In other words, humans live in an intentional *Umwelt*. Interactional properties such as colors or propositional attitudes need competent perceivers and causal properties in order to be said to be objective. Thus, reasons are real properties, such as colors are real. But they are not real in the same sense we can say that physical entities, such as wavelengths for instance, are real; The latters do not need to be observed to exist.

## Reasons, Causes and Real Patterns

Let’s consider two implications for physicalism related to the IS. According to eliminativism, we can imagine that a mature psychology will eradicate folk psychology. Neuroscience might be, someday, in measure to provide a mature psychology able to explain and predict behaviors more accurately. Elimininativists bet on such a theoretically possible future that would allow creating a propositional-attitude-free theory on the basis of which behaviors could be explained and predicted. In doing so, they presuppose the physicalist Laplacian dogma (logical determinism): if we could know all the laws of the universe, including the laws of the brain, then, we could predict others’ behaviors from the current physical state of the world. The fact is that, even before asking whether it is conceptually possible – and always answered with the irrefutable “in-principle-if-not-in-practice” mantra – we have to acknowledge that current neuroscience does not dispose of such knowledge, nor of the calculating powers it might necessitate. The point is that folk psychology, at least in comparison with what neuroscience and physics can currently predict, is still the most reliable and easy way to explain and predict intentional actions in everyday life. But does this mean that folk psychology can claim a quasi-scientific status autonomously, i.e., without reference to the causal phenomena neuroscience and physics deal with?

The IS does not invalidate physicalism (i.e., that physical ontology and natural causality underlie phenomena, including mental ones), but disputes only the claim that physics would be the only level at which one could successfully predict behaviors. Consider the following example: when bees die (Dennett, 1983), their congeners remove their dead bodies from the hive. A naïve observer or a realist such as Grünbaum, might observe the scene and successfully proposes to put apiary behavior into the mold of the practical syllogism, according to which the desire-cum-belief can be identified as the reason of an action Grünbaum: bees desire to avoid diseases, and they believe putrid bodies to be the vehicle of diseases, and thus they remove dead bodies from the hive. The descriptive practical syllogism can be turned into a conditional or predictive one, which will prove resistant to falsification. Then, the intentional competent observer has brought to light an objective pattern or behavioral regularity, which has been confirmed by recurrent facts.

But a “kill-joy” explanation has in fact shown that bees’ behavior can be more economically explained as a tropism. Dead bees secrete oleic acid. As it turns out, any object impregnated with oleic acid, including live bees, is removed from the hive. Recall Dretske’s teleosemantic theory (Dretske, 1988). The presence of a moving black spot triggers the frog’s reflex of tongue-catching. Even if the neural mechanism plays a functional role – it triggers the fly catching reflex devoted to survival purposes – the internal cerebral state does not indicate the fly *per se*, but instead a black spot moving (Lettvin et al., 1959). The bee’s oleic blind detector plays the same function. The point is that the psychological description – the bee representation about dead bees, the desire to keep healthy, etc. – is quite false. There is nothing like propositional attitudes in it, not even an isomorphic pattern in the brain (Fodor’s mentalese, for instance), yet only blind detectors triggering motor procedures. In other words, the ascribed reasons – the desire-cum-belief – works, even if it is false. But it works not because reasons are causal *per se*, but because it captures objective behavioral regularities, causally determined by inner blind regulation traits. Consequently, Grünbaum can claim that the desire-cum-belief X makes a difference in the occurrence of the bee’s behavior Y. But the desire-cum-belief is here wrongly identified as the causal factor.

In sum, the IS emphasizes the objectivity of the processes captured by folk psychology, provided that the predictions that it generates are successful. This conclusion does not contradict the fact that the patterns are in the eye of the beholder, nor with the faulty interpretations it might give. Thus, if it challenges the predictive pretentions of the reductive programs of neuroscience and physicalism (at least their current pretentions), the IS theory has in common with the latter the assumption according to which psychological states rest on blind (non-intentional) mechanisms. Thus, while eliminativism calls for folk psychology to be abandoned because of it inaccuracy or its falsity, the intentional stance is richer, specifying how folk psychology, in spite of its defects, can be said to be a scientific tool. It is if it enables heuristic explanations that are empirically testable in the light of publicly observable behaviors.

This point has to be emphasized contra the relativism that weakens the hermeneutics as well as the extensionist approaches. As a matter of fact, people in their daily life are successful in using folk psychology. But if such successes do not necessitate to be scientifically evaluated in daily life, what allows saying these behavioral regularities are objective, rests on the fact that if such predictions are *really* successful, they must be, in principle, “testable by standard empirical methods of variable manipulation” (Dennett, 1991). We will see that extensionists are precisely missing their point in eluding the necessity to undergo scientific procedure in order to validate reasons ascriptions.

## Facts of Interpretation or Neuro-Hermeneutics

The IS can be said to be a hermeneutics that, as a consequence of the inscrutability of the reference, opens a room or allows that several interpretations might be good candidates for explaining the same behavior, be it that they refer to different interpretative schemas (psychoanalysis, astrologer, etc.) or to different psychoanalysts. Note that even if one can easily identify the reasons that may motivate simple actions, as in the paradigmatic case of the bees that remove dead bee bodies, the more the behaviors are complex, the more the primary reasons are hard to isolate. This is precisely what psychoanalysis makes evident. The kind of irrational states psychoanalysis deals with necessitate building up special stories with multiple and sometime contradicting reasons. What makes the IS interesting is that it does not matter if we can multiply the interpretations if we can count on a good pragmatic criteria allowing us to select the relevant ones. Thus, while the acausal-hermeneutics uses the causes-reasons distinction as a justification for the understanding’s epistemic autonomy toward explanation, the IS constructs a causal-hermeneutics, since reasons are articulable and evaluable in reference to the causal factors that underlie them. Both the inner causal features and the measurable behaviors represent, therefore, the kind of interpretation facts one can lean on to validate the heuristic value of a reason ascriptions.

Let’s emphasize the heuristic role of interpretation in discovering the inner causal features (such as the oleic acid detector) that determinate the behavior. As Dennett puts it:

“It is not that we attribute (or should attribute) beliefs and desires only to things in which we find internal representations, but rather that when we discover some object for which the intentional strategy works, we endeavor to interpret some of its internal states or processes as internal representation. What makes some internal feature of a thing a representation could only be its role in regulating the behavior of an internal system” (Dennett, 1981).

The last part of the quote is important here. In the causal-hermeneutical understanding of the IS, intentional attributions are not only evaluable in the light of the successful behavioral predictions it is possible to infer from them. The IS implies that if a mental state indicates an internal regulating feature, any accurate interpretations *ought* to be correlated to a regulating causal feature too.

Let’s transpose this to neuroscience and to psychoanalysis (not to say to neuropsychoanalysis). Neuroimaging techniques are now appropriate for testing these kinds of alternative predictions. We can expect that subjects’ brains might be sensitive or might get active when presented with stimuli linked to their putative ascribed reasons. Such a possibility would be even more plausible if the kind of ascribed reasons are such as to refer to intentional ascriptions the subject would ignore, such as the unconscious mental states psychoanalysis claims to be able to discover thanks to its interpretation schema. Consider the following experiment by Shevrin et al. (1996).

Shevrin et al. (1996) conducted an experiment with subjects suffering from neurosis. On the basis of a couple of tape-recorded interviews, clinicians constructed a list of affect-laden words which, according to the Freudian theory, were supposed to refer to the patients’ unconscious conflicts^6^. It was counterbalanced by another list the words of which were chosen by the patients in reference to their subjective (conscious) apprehension of the constituents of their inner conflicts^7^. A third list of universal affect-laden words constituted a control condition.

The three lists were presented to patients in two different conditions, subliminally and supraliminally, while the subject’s ERPs (evoked-response potential) were recorded. The results show different patterns of responses triggered by subliminal and supraliminal exposure. On a one hand, the words chosen by the analysts, presented subliminally, triggered a specific time-frequency pattern: the highest frequency has the shortest latency. The reverse was true for the patient’s words presented subliminally. In the supraliminal condition, the words chose by the analyst and that had the lowest frequency now had the shortest latency, while the reverse was true for the patient’s words. In others words, the results suggest that the two kinds of words were associated and processed differently in comparison to the control condition where the subliminal-supraliminal presentations did not displayed inverse patterns.

What can be inferred from this? We could expect that if the “psychoanalytic stance,” as we will call this version of the IS for the moment, were not efficient at capturing internal features or neural patterns, it would have never induced patients’ brain specific reactions in comparison to control words choose randomly. Of course, the fact that these neural features are triggered does not imply that the unconscious reasons provided by the psychoanalysts are the *real* cognitive or unconscious mental determinants of the patients’ neuroses. But these words were chosen within the strict limits of an interpretative schema. Moreover, they were not chosen with the patients – who thus were not aware of them as mental determinants – and were not perceived consciously in the subliminal condition. In other words, they are pure interpretative material. Thus, if we take for granted that the experiment’s protocol suffers from no methodological deficits, we could predict that if the interpretations were not relevant, it would have never triggered specific neural patterns. In other words, in line with the causal-hermeneutical framework – in this case we can talk about neuro-hermeneutics – we can claim that if the key words, related to reason ascriptions, do trigger neural patterns, we do possess, contra Grünbaum, a retrospective validation of their heuristic value.

## Conclusion

Let’s go back to the initial question: is psychoanalysis an extension of folk psychology? I will give two answers. Psychoanalysis surely is an extension of the folk psychology range of application. But the term “extension” cannot be invoked as a means to defend psychoanalysis’ autonomy, taken that it fails at overcoming the problems it was supposed to solve: relativism, acausality, absence of proof. In a sense, I propose here to transform the initial question into these terms: is the intentional stance an extension of folk psychology? As a matter of fact, the IS is a theory that specifies the scientifically credible use of folk psychology as a heuristic tool, and the kind of criteria that allow us to evaluate its accuracy in certain given instances. As a scientific extension of folk psychology, it comes more than what is required to the daily life folk psychological ascriptions. If the folk psychology does reveal objective patterns, the latter have to be, in principle, testable by standard empirical methods of variable manipulation. Thus if psychoanalysis is an extension of folk psychology, “extension,” here, has to be understood in a stronger sense than the extensionist’s use of the term: as a specific use of the intentional stance, psychoanalysis must be a *scientific extension* of folk psychology. Thus, in line with Grünbaum it must accept the tests of scientific criteria, e.g., variable manipulation, statistics, EEG, groups control, etc^8^.

The problem is that extensionists often write as though psychoanalysis has no scientific pretentions and is nothing more than a naïve use of folk psychology. But Folk psychology cannot be in the same time what has to be proved to be accurate and what is convoked as a control of what is accurate. This is precisely one consequence of the non-equivalency of causes and reasons. If reasons are essential as heuristic ascriptions and do reveal something about the regularities underlying behaviors, they are not causal *per se*; instead, only the behavioral regularities and the inner regulation features it indicates are causal. These can at least be accounted as facts of interpretation. In decoupling reasons and causes, the IS allows rehabilitating the interpretation theory provided by hermeneutics, yet overcomes its deficiencies. In other words, the IS can be qualified as a causal-hermeneutics.

Lastly, Shevrin et al.’s (1996) seminal experiment can be seen as a brilliant example of how psychoanalysis might become, among the plethora of interpretative schemas, a promising hybrid technique devoted to enlightening interpretative facts. For instance, if classical cognitive science consists in investigating universal cognitive mechanisms in manipulating universally intelligible stimuli, Shevrin’s experiments open an avenue for studying universal cognitive patterns by the means of idiosyncratic ones. In others words, it uses the IS to catch the objective bases of “idiosyncratic biases.” Another important point is that Shevrin’s causal-hermeneutics, that I defined more precisely as a neuro-hermeneutics, is not possible without taking seriously a scientifically exploitable contrast between first-person and third-person perspectives. In a way, then, neuro-hermeneutics might represent a novelty within the tool-box of neuroscience, especially in social cognition research.

But the most important implication of this experiment concerns the kind of answer we can give to the initial question: is psychoanalysis an extension of folk psychology? The point is that, on a one hand, Dennett shows to what extent there are facts of interpretation that can be credibly evaluated, compared, selected, and rejected. On the other hand, Shevrin’s experiment, in spite of its defects, shows how we can really turn the interpretation issues into testable ones. Why am I including the caveat, “in spite of its defects”? Indeed, if the experiment provides evidence that we can, adopting a third-person perspective, ascribe accurate unconscious reasons, under no circumstances does it give us any proof that the psychoanalytic stance is required in order for these neural regularities to be detected. To prove that, the neural effects imputed to the analysts’ list should not only be compared to arbitrarily chosen affect-laden words, but to alternative lists. For instance, Shevrin should have compared the list a psychoanalyst would have elicited, in reference to a patient, to the one that another psychoanalyst would have constituted, to the one given by a cognitive-behaviorist psychologist, an astrologer, a Chinese physician, an ordinary person… Why an ordinary man? Because this is the emblematic competent folk psychologist. If the difference between what I called the weak and the strong versions of extensionism is well founded, the folk psychologist’s and the psychoanalyst’s lists should trigger different neural patterns. On this basis, we can imagine many others controls allowing neuropsychoanalysts giving a fine-grained and experimentally motivated, answer to the initial issue.

## Conflict of Interest Statement

The authors declare that the research was conducted in the absence of any commercial or financial relationships that could be construed as a potential conflict of interest.

## Footnotes

^1^The kind of naturalism that I endorse here posits that any kind of phenomena is underpinned by natural causes and has to be explained in reference to the best science we have at our disposal. Such a position does not imply eliminative materialism, i.e., that intentional phenomena would play no role in scientific inquiries.

^2^The term hermeneutics comes from the Greek *Hermeneuein* that means explaining, interpreting, translating.

^3^On the recent debates on whether it is conceptually possible for a representation to be unconscious, see Searle (1992) and Smith (2004).

^4^Such a holistic coherence is nothing more than an alternative formulation of the hermeneutics circle that posits that the understanding of a text as a whole is established by reference to the individual parts and reciprocally.

^5^The exact words are: “Exoriare aliquis nostris ex ossibus ultor!” The scholar starts dividing the word into “a” and “liquis.” To Freud, the term liquid is linked to “exoriare” (originating) that is associatively related to “excreting” or “expelling.”

^6^Some might claim that this kind of interpretation does not take into account the dialogic or interpersonal nature of interpretation. The analysant’s states of mind is not what has to be “discovered.” In line with the hermeneutic tradition, the interpretation should rather be conceived of as emerging from the relational interpretative process. I totally agree. But this does not forbid the psychoanalyst to provide an interpretation as if it was third-person knowledge, abstracted from the relation, and that would take the patient’s psychic determinants as the object of the psychoanalytic inquiry.

^7^To give an example, if the “aliquis” scholar had participated to this experiment, the terms “liquid” or “period” should have been part of the analysts’ list. As the scholar attributed his lapse to fatigue, “tiredness” could have been added to his own list. For a comprehensive description of the experiment, including the clinical details, see Shevrin et al. (1996).

^8^One of the reviewers relevantly pointed out that Grünbaum’s validation criteria, and thus mine, are only appropriate in the limits of the positivist framework he adopts. There would be no reason, except a “positivist” bias, to privilege objective facts better than the interpretation internal consistency. Hermeneutics would not be committed to relativism, taken that the interpretative process is always constrained by contextual guides (Stern, 2012). It is true that my naturalist commitment implies verificationism, i.e., that experimentation only should allow overcoming the hermeneutics limitations? But my claim is not that hermeneutics runs the risk of relativism. On the contrary, the bee example shows that the plurality of ascribed reasons is constitutive of the interpretative process and that the latter is embedded in the context (interpersonal, social, historical, etc.). Nevertheless, interpretative plurality does not imply that there are no interpretative facts. My goal is not to report hermeneutics relativist risks, but rather its acausalitity.
